# JAGRITI (ಜಾಗೃತಿ) - A study on enhancement of health communication for sickle cell disease (SCD) among Soliga Indigenous community in Chamarajanagar, Karnataka: Study protocol

**DOI:** 10.12688/wellcomeopenres.23935.1

**Published:** 2025-07-15

**Authors:** Praveen Srinivasa Rao, Padmakumar K, Achyutha Nagaragadde, Deepa Bhat, Yogish Channa Basappa, Tanya Seshadri, Manjula Venkataraghavan, Upendra M Bhojani

**Affiliations:** 1 Centre for Adivasi Health, Public and Policy Engagement Unit, Institute of Public Health Bengaluru, Bengaluru, Karnataka, 560070, India; 2 Manipal Institute of Communication, Manipal Academy of Higher Education, Manipal, Manipal, Karnataka, 576 104, India; 3 Centre for Commercial Determinants of Health, Institute of Public Health Bengaluru, Bengaluru, Karnataka, 560070, India; 4 JSS Medical College, JSS Academy of Higher Education and Research, Mysuru, Karnataka, 570015, India; 5 Family Medicine and Population Health, University of Antwerp, Antwerp, 331, Belgium

**Keywords:** Sickle Cell Disease, SCD, Haemoglobinopathies, Health Communication, Community Engagement, Indigenous, Tribal Population, Health information, Awareness, Community engagement, Soliga community, Health Education, Adivasi community

## Abstract

**Background:**

Sickle Cell Disease (SCD) is a genetic disorder that disproportionately affects Indigenous populations. Tailored awareness and management strategies are crucial for reducing the prevalence of SCD and improving its management. Existing awareness campaigns are inadequate and are often devoid of cultural appropriateness, local language, and evidence-based content/design. There is a dearth of knowledge on what constitutes effective health communication for these communities.

**Objectives:**

This study aims to examine existing SCD-related knowledge, its sources, and the health communication-supportive infrastructure among the Soliga community in the Chamarajanagar district of Karnataka. It will identify any established health communication strategies used for and/or by Indigenous communities, assessing their strengths and limitations. Additionally, the study seeks to understand community preferences for health communication, including language, content, and delivery. Insights from these findings will guide the development of a comprehensive and practical health communication guide for SCD.

**Method:**

The study employs a mixed-methods approach following the triangulation design by Creswell and Plano Clark (2007). The study begins with a community-based survey to assess existing SCD knowledge, information sources, and communication-supportive infrastructure within the Soliga community. Next, a scoping review will identify global health communication strategies for Indigenous populations, supplemented by Focus Group Discussions with community stakeholders to explore preferences for culturally appropriate communication methods. The insights from these stages will guide the development of a comprehensive, evidence-based guide for effective SCD-related health communication tailored to Indigenous populations.

**Discussion:**

This study explores ways to advance culturally appropriate, evidence-based health communication strategies. It will address the knowledge gap in effective SCD communication for Indigenous communities by emphasising the role of community preferences. This study's outcomes aim to guide future health communication campaigns, ultimately enhancing SCD awareness and management among Indigenous populations.

## Plain Language Summary (PLS)

**Study title:**

Health Communication for Sickle Cell Disease(SCD) Among Soliga Indigenous Community in Karnataka: Gaps, Preferences, and Effective Strategies

**Background:**

SCD is a severe hereditary disorder affecting a large portion of India's Indigenous communities, leading to high morbidity and mortality rates. Although the Indian government launched the National Sickle Cell Anaemia Elimination Mission in 2023, awareness about SCD remains low among Indigenous populations. One reason for this is the lack of evidence-based health communication strategies tailored to community preferences. This gap makes it challenging to raise awareness and promot better management of SCD.

**Why this study?:**

To reduce the prevalence of SCD and improve health outcomes, raising awareness about the disease is essential. However, for health messages to be effective, they must be designed and delivered in ways that resonate with the community's needs and preferences. This study focuses on the Soliga community in Karnataka to understand their current knowledge about SCD, their sources of health information, and their preferences for how health messages are designed and communicated. By identifying these aspects, the study aims to design better health communication strategies tailored to the community. The study will use a mixed-methods approach, including Surveys, Scoping Review, and Discussions with community members.

**How this study will help:**

The findings from this study will guide the development of a comprehensive communication guide to improve SCD awareness among the Soliga community. By aligning health messages and campaigns with community preferences, healthcare providers and policymakers can communicate more effectively and respectfully. Although the study is focused on one Indigenous community, its insights could help design health communication strategies for communities with similar characteristics. Ultimately, the research aims to enhance SCD awareness and care, contributing to better health outcomes for Indigenous populations.

## Introduction

Indigenous communities, also termed as Scheduled Tribes or Adivasi, constitute 8.6% of India’s total population. We use the term 'Indigenous communities' in this paper considering its common usage and shared understanding in international literature. In India, they are commonly referred as tribal communities in official parlance wherein the Indian constitution recognises 'Scheduled Tribes' as a category referring to these special groups and there are 'tribal welfare' departments (government agencies with a mandate to promote welfare of these communities) ( Ministry of Tribal Affairs, Government of India, n.d.). The Expert Committee on Tribal Health Report mentions 705 Indigenous communities spread across the country, primarily living in rural areas ( Kshatri *et al.*, 2023; Report of the Expert Committee on Tribal Health, 2018). Their distant location and inadequate infrastructure severely hamper healthcare delivery and access among these populations. These communities are frequently found in isolated, difficult-to-reach places, such as steep terrain, forests, or areas with poor transportation ( Deb Roy *et al.*, 2023). Health indicators among Indigenous people are comparatively worse than those of the general population ( Report of the Expert Committee on Tribal Health, 2018). The lack of health education further exacerbates this situation ( Anderson *et al.*, 2016; Islary, 2014). The health condition of Indigenous communities in Karnataka is no different. The Indigenous population constitute 6% of the state population in Karnataka ( Ministry of Tribal Affairs, n.d.)

Among the many severe health conditions ailing Indigenous communities, Sickle Cell Disease (SCD) is one of the leading health ailments. In India, sickle haemoglobin was first identified in 1952 among the Indigenous communities of the Nilgiris ( Lehmann & Cutbush, 1952). Childbirths with SCD and the prevalence of SCD are high among Indigenous communities as compared to the general population in India. According to the Health Ministry report, this hereditary disorder affects a large portion of India's Indigenous people; around 1 in 86 ST newborns have SCD ( Ministry of Health and Family Welfare. Government of India, 2023). It affects patients throughout their lives ( Surti *et al.*, 2024). Vaso-occlusive crisis, acute chest syndrome (ACS), stroke, acute renal failure, splenic sequestration, and acute vision disorders are examples of acute comorbidities that may develop at any age. As people age, they are more likely to develop chronic comorbidities such as gout, diastolic heart issues, pulmonary hypertension (PH), leg ulcers, end-stage renal disease (ESRD), and visual problems ( Ogu & Billett, 2018). Around 1.80 crore Indian Indigenous people are expected to have sickle cell trait, and 14 lakh have SCD ( National Health Mission, 2016). To reduce the incidence, morbidity, and mortality of SCD, the World Health Organization has also recognised SCD as a public health issue and recommended more information-sharing and awareness-raising campaigns tailored to specific socioeconomic, cultural, and health system contexts ( World Health Assembly, 2006). SCD is one of the ten unique health issues that disproportionately affect Indigenous population, according to the Ministry of Health and Family Welfare tribal health expert committee study ( NSCAEM, 2023).

Awareness creation is an essential aspect of any human development activity. In achieving better health outcomes, communication of relevant information with all stakeholders is vital ( Lkreps, 1988). Communication in the health sector has been a dynamic and varied process involving a range of interactions between patients, medical professionals, and the general public ( Gianfredi *et al.*, 2018). Seeking, processing, and disseminating health information is mainly accomplished through human communication. However, because communication is widespread, omnipresent and ambiguous in the healthcare industry, important communication activities are sometimes taken for granted, and the nuances and complexity of health communication are frequently overlooked, neglected, and poorly used ( Lkreps, 1988). 

As scholars and practitioners emphasise, health communication plays a vital and pivotal role in addressing global health challenges. To comprehend human behaviour in this context, researchers increasingly focus on theoretical frameworks exploring how risk perceptions, social norms, emotions, and uncertainty influence health behaviours ( Rimal & Lapinski, 2009). Evidence shows that the successful implementation of awareness programs has contributed to a notable increase in couples opting for prenatal diagnosis for SCD in India ( Colah *et al.*, 2014). The role of health communication is crucial in the prevention, management and treatment of SCD among Indigenous population. Since it is an approach that raises awareness, adequate knowledge about the disease helps reduce its prevalence and improve adherence to treatment by assisting people in making informed decisions. Also, very few studies and literature have focused on the importance of health communication in addressing sickle cell disease in India ( Surti *et al.*, 2024). The National Sickle Cell Anaemia Elimination Mission by the Government of India emphasise the need for awareness generation as a foundational strategy ( NSCAEM, 2023). Since the mission is in its early stages, it is essential to generate evidence-based health communication for SCD, keeping in mind the preferences of communities. India has demonstrated a successful history of campaigns for health communication, ranging from ailments like HIV/AIDS, TB, and Polio as well as reproductive and sexual health ( Mirchandani & Chitrapu, 2023). 

Restricted access and lack of accurate health information contribute to individuals struggling to make informed decisions about their health ( Soman *et al.*, 2023). A news article published in 2012 by the World Bank also supports the narrative that the lack of health awareness among Indigenous communities leads to delays in seeking medical care. Often, most health awareness campaigns are designed by the medical community and not the communication experts, and they lack effectiveness and comprehension due to poor and un-tested health messages ( World Bank News, 2012). Often the diversity in socio-cultural practices, languages of communities, rituals and varied customs hinder the effectiveness of health communication and service delivery ( Soman *et al.*, 2023).

Earlier studies point to generally low levels of awareness about SCD among indigenous communities. Also, awareness level varies across geographical sites in India. The highest reported in the district of Gujarat was 78.4%, and the lowest was among the districts of Assam, at 1.1%, and 10.9% in Karnataka and Madya Pradesh. The district in Karnataka, which has a 10.9% reported awareness level, is adjacent to our study district. It also points out that the two major sources of information about SCD include health workers and relatives/friends. The usually expected sources, such as mass media (TV/radio) and awareness programs, were cited by less than 2% of people as sources for SCD-related information ( Babu *et al.*, 2022). These findings imply a massive need for appropriate communication on SCD-related issues for Indigenous communities in ways that are acceptable to them. Considering the burden of SCD in India, the Government of India in 2023 launched a new Mission to address SCD, aiming to create more awareness, proper diagnosis, and effective disease management to reduce the future prevalence. Along with the other measures, awareness through evidence-based, culturally tailored community-level awareness programs using various materials and channels on symptoms, diagnosis, management, and preventive measures have a more significant role in increasing awareness at the grassroots level ( Aggarwal & Bhat, 2023).

Therefore, this research study aims to identify the gaps in the current health communication on SCD at the community level and understand the community's preferences regarding the content and delivery of SCD-related health information. Based on these insights, the study will develop a comprehensive guide to assist in designing and delivering effective SCD communication campaigns.

### Objectives

The study has four objectives as outlined below;

1. To describe the current state of SCD-related knowledge, sources for such knowledge, and health communication-supportive infrastructure among Soliga Indigenous community residing in the Chamarajanagar district of Karnataka.
2. To identify any established health communication strategies, including their strengths and limitations, for effective health communication used for and/or by Indigenous communities.
3. To identify community-preferred and culturally appropriate methods (language, content, delivery) for SCD-related health communication among Soliga communities residing in the Chamarajanagar district of Karnataka.
4. To design a comprehensive guide for effective health communication related to SCD

## Methods

### Study design

We propose a mixed-methods study to identify gaps and opportunities for improving SCD-related communication. Specifically, we will use a triangulation design one of the four mixed-methods designs (Creswell and Plano Clark (2007), as cited in Clark *et al.*, 2008). This design facilitates cross-validation of results from quantitative and qualitative components making the study findings more robust and credible. The qualitative data will provide in-depth contextual insights into community perceptions, cultural influences, and communication preferences, while the quantitative data will measure SCD awareness levels, sources of information, and the current communication infrastructure. Using this approach will help to develop a comprehensive SCD communication guide, ensuring it is evidence-based, culturally relevant and well-informed by multiple perspectives. The study follows three key stages, with participants and outcomes detailed at each stage.

**Figure 1.**
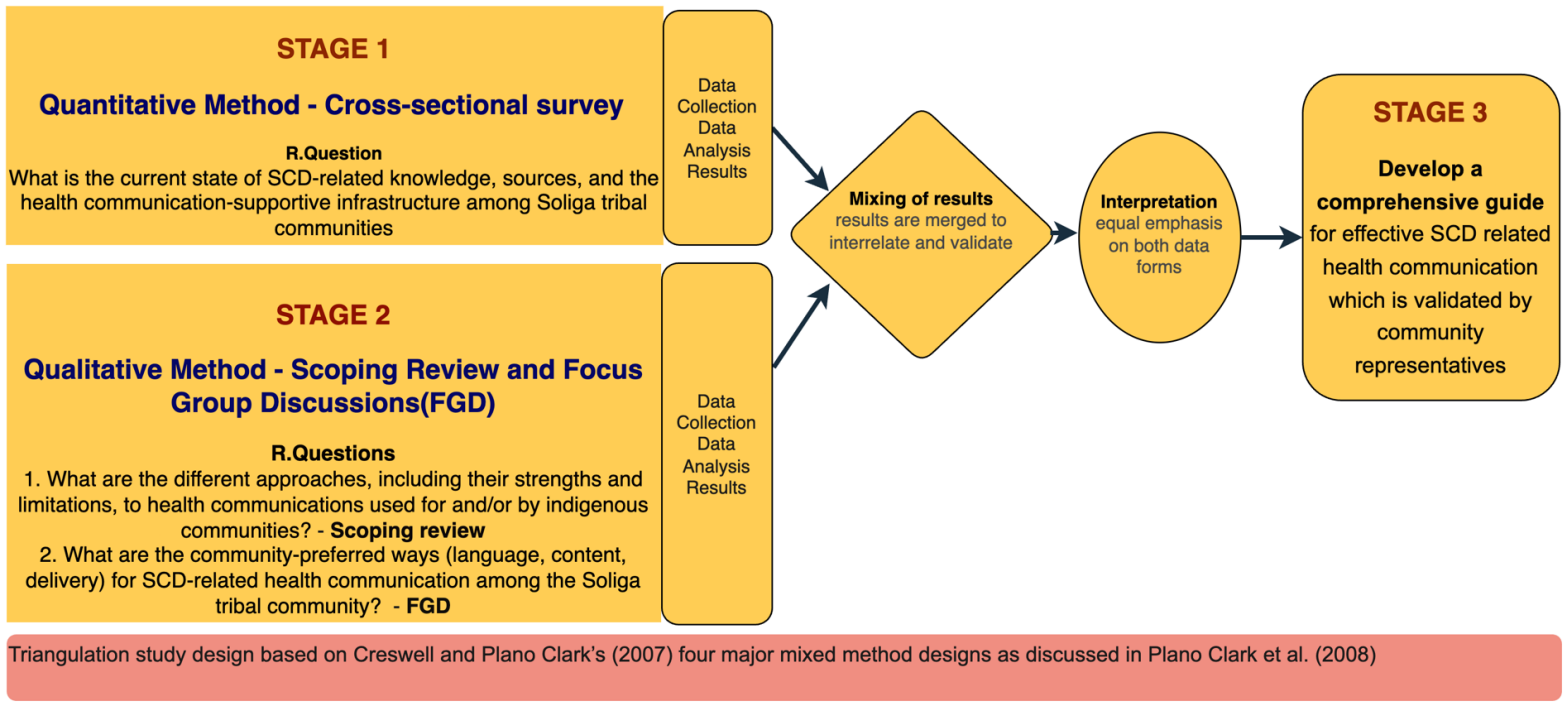
study stages and design explained - Triangulation variant of the mixed methods design.

In **Stage 1,** we will conduct a cross-sectional survey to assess the current knowledge about SCD, understand the sources of health information related to SCD and available health communication supportive infrastructure among Soliga Indigenous community residing in the Chamarajanagar district of Karnataka. In **Stage 2**, a qualitative approach will be employed, where we will conduct qualitative enquiry through a scoping review to understand existing health communication among Indigenous population for the different methods, their strengths and limitations used by or for Indigenous communities. Additionally, as part of stage 2, Focus Group Discussions (FGDs) will be conducted with people aged 18 to 40 in the Soliga community. These discussions aim to explore community-preferred ways and methods of health communication. Stages 1 and 2 will inform **Stage 3**, wherein a comprehensive guide will be developed. The guide will compile all necessary information and key elements to design effective health communication strategies for the Soliga community and other similar Indian Indigenous communities regarding SCD ( Figure 1).

### Study setting

The study site in this study is rural parts of Chamarajanagar district located in the southern part of Karnataka state in India, focusing on the Soliga population of the district.

**Figure 2.**
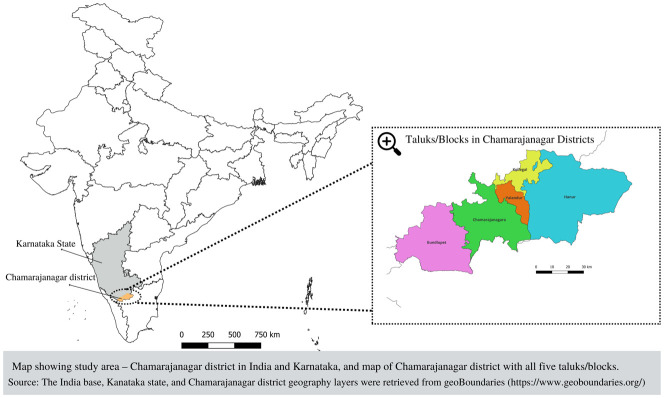
Map showing study area – Chamarajanagar district in India and Karnataka, and map of Chamarajanagar district with all five taluks/blocks. (The India base, state, and Chamarajanagar district geography layers were retrieved from https://www.geoboundaries.org/ ( Runfola *et al.*, 2020)

***Site profiling.*** Karnataka is home to over 42 lakh Indigenous people, accounting for 6.95% of the state population. The government of India has identified as many as 50 scheduled tribes in Karnataka, 14 of which are predominantly residents of the state ( Roy *et al.*, 2015). Chamarajanagar district, located in the Karnataka-Tamil Nadu-Kerala trijunction ( Figure 2) in south Karnataka, is renowned for its lush forests, rich Indigenous heritage, and 48% forest cover. The district's total population, as per the census 2011, is 10,20,791, with a literacy rate of 61.43%. There are five taluks/blocks in Chamarajanagar, namely Chamarajanagar, Kollegal, Hanur, Yelandur and Gundlupet. District Tribal Welfare Department (DTWD) data mentions 158 hamlets (Indigenous settlements), in the district. 

***About Soliga.*** Soliga community is one of the forest-dwelling scheduled tribes in Karnataka with deep-rooted ties to the land. Soliga is also spelled as Soligaru, Sholiga, Sholigaru, Solega, Sholaga ( Koppad *et al.*, n.d.). The total population of the Soliga community is 33,819 in the state, present in three districts – Chamarajanagar, Mysore and Madya – but most of the population resides in Chamarajanagar district. The population of the Soliga community in Chamarajanar is 18,163 as per the 2011 census. They have a dialect called Soliga Nudi ( Morlote *et al.*, 2011).

The following section outlines the study stages in detail.

## Stage 1

### Quantitative study - survey research

***Rationale.*** This methodology was adopted to thoroughly analyse SCD-related information, sources, and available communication-supportive infrastructure among the study population. This method ensures that varied geographical and population characteristics are captured. This approach offers a thorough understanding of SCD-related knowledge in the regional setting that guides specific actions and policies.

***Sample size and sampling strategy.*** Given that 10% of SCD-related knowledge is in Mysore district ( Babu *et al.*, 2022), a neighbouring district of Chamarajanagar, we hypothesised a prevalence of SCD-related awareness level of 15%. We plan to collect 428 samples by assuming a 5% alpha error, a 95% confidence interval, a design effect 2, and a non-response rate of 10%.

***Sample strategy.*** We will use a multi-stage stratified random sampling technique. In the first stage, we will select all five taluk/blocks from Chamarajanagar district. Within each selected taluk/block, we will utilise the DTWD data to randomly draw one-third of hamlets using a simple random sampling technique in MS Excel. Finally, the number of participants to be sampled within each selected village will be proportionally allocated based on population size. In case of inadequacy in sample size at the hamlet chosen, we will collect it from the nearest hamlet (in terms of distance). In each village, we will collect data from all directions starting from a central point, such as a temple, school, or community hall. After discussing with the head of the household, we will invite an eligible household member to participate in the study.

***Participant selection and allocation.*** SCD is a genetic condition, and the lack of awareness about it during reproductive age significantly contributes to the increasing prevalence of SCD traits and the disease within the community. Therefore, we will select the Soliga population residing in Chamarajanagar district, from all genders and a range of age groups between 18 to 40, representing the diversity within the community.

***Participant recruitment (Inclusion and exclusion criteria)***

**Inclusion criteria:**

1. Participants belong to only the Soliga community and reside in the Chamarajanagar district.
2. Participants between the ages of 18 and 40 years old.
3. Participants who provide informed consent

**Exclusion criteria:**

1. Individuals who are unable to understand the survey questions due to mental health issues, language issues, or speech or hearing loss
2. Participants under the influence of alcohol (While we do not have a precise mechanism to confirm alcohol influence during data collection, participants will be excluded based on the data collector's observation and the participant's self-report of alcohol consumption).

***Procedures for data collection.*** As a responsibility, gesture of respect, and community engagement, local community leaders who serve as influential figures/gatekeepers within the community will be approached to explain the study and seek their support before the commencement of the study. Their endorsement can facilitate acceptance and cooperation from community members.

Participants will be identified based on predefined inclusion and exclusion criteria. A participant information sheet will convey the study information to participants, ensuring cultural sensitivity by aligning with the community's language and communication preferences. We will obtain informed consent from participants before collecting data. Trained data collectors, recruited from the same Indigenous community (Soliga), will administer the structured questionnaires in person at the participant’s place of residence by reading out the questions, clarifying any doubts, and recording the responses on a mobile device using the KoboToolbox data collection application.

***Data collection tools.*** Data will be collected using preloaded handheld devices (mobile/tab) in Kobo Toolbox web application, an open source data collection tool. The questionnaire has been developed based on guidance from an earlier study by ICMR conducted in Mysore and other sites ( Babu *et al.*, 2021). The questionnaire has been further customised to suit the study requirement ( annexure 1).

***Consent process.*** Trained data collectors will conduct the informed written consent process before data collection. Participants will be approached individually in familiar community settings, ensuring privacy and confidentiality. The data collectors will use the information sheet to explain the study's purpose, procedures, and potential risks and benefits. Participants will be assured that their participation is voluntary. Consent will be documented through signed consent forms, with copies for participants' records. In case a participant is not able to read and comprehend, data collectors will read out the consent process and about the study and, upon agreeing, will take a thumb impression of the participant in the presence of a witness.

***Data analysis.*** For the quantitative survey, we will analyse the data using descriptive and inferential statistical methods. We will summarise the data using descriptive statistics such as frequencies, percentages, means, and standard deviations. Cross-tabulations will examine the relationships between demographic variables and SCD awareness. Inferential statistics, such as chi-square tests, will be conducted to explore associations between key variables like knowledge levels and the use of specific health communication channels. Data will be processed and analysed using the statistical software R. All results will be presented in tables and graphs for clear interpretation. We will further elaborate on significant findings to inform the design and development of the guide.

## Stage - 2

Qualitative study – scoping review and Focus Group discussions (FGDs)

Following stage 1, we will proceed to stage 2, which is qualitative data collection and analysis. We will further divide this stage into two inquiries, namely:

### Scoping review

### Focus Group Discussion (FGD)

### Stage 2.1 Scoping review

We will conduct a scoping review to identify established health communication strategies, including their strengths and limitations, for effective health communication used for and/or by Indigenous communities.

***The rationale for the method.*** This approach will help understand existing ways of health communication among Indigenous population across the globe. The scoping will also help synthesise best practices that various stakeholders have adopted in addressing health communication in different demographics.

***Methods.*** The scoping review will follow Arksey O'Malley's methodological framework ( Arksey & O'Malley, 2005) involving five stages – Identifying the research question, selecting relevant studies, selecting data, charting and summarising the results. Electronic databases, namely PubMed, Web of Science and Scopus, will be utilised. We will also do citation tracking as a supplement search. 

***Eligibility criteria***

**Inclusion criteria**

1. Literature published with a focus on Indigenous communities across the globe. The articles must contain health communication strategies, programs, campaigns, or approaches to the Indigenous population
2. Literature published in English
3. Articles published in peer-reviewed scientific journals

***Search strategy.*** We will use a combination of keywords related to Indigenous communities and health communication for the search strategy. We will tailor the search strategy to each database using relevant Boolean operators while generally following the structure. We will identify search words based on the Population, Concept, and Context (PCC) framework applied to research questions.

***Data sourcing.*** Rayyan (free version), a web-based software, will remove duplicates and manage the articles. Two reviewers will independently review the titles and abstracts for sourcing and document the reasons for exclusion. We will match the shortlisted results, and if there are differing decisions, a third independent reviewer will assist in building consensus. We will conduct a full-text screening of the shortlisted publications.

***Data charting.*** We will use a standardised data extraction form (in MS Excel) to collect relevant information from the included studies. After completing the full-text review, a researcher will extract data and create a data chart. A second reviewer will examine a subset of papers (one out of ten) to verify the extracted data. Both researchers will then review the extracted data and resolve any issues or discrepancies. A third independent reviewer will assess the articles if they cannot reach a consensus. The data will be presented using a PRISMA flow diagram ( Page *et al.*, 2021).

***Data analysis.*** The characteristics of the included studies will be summarised, and common themes, strengths, limitations, and relevant thematic analysis will be outlined. Themes will focus on previously attempted methods, strategies, and approaches for health communication, what worked and did not work, challenges encountered, campaign design, and any other emerging themes during data extraction.

### Stage 2.2 Focus Group Discussion (FGD)

Focus Group Discussions will be held with Soliga community members to understand communication preferences.

***Rationale for study design.*** Guided by the scoping review, we will conduct FGDs to understand community preferences in health communication among the Soliga community. FGDs are more appropriate as they facilitate group interaction, essential for capturing collective perspectives, social norms, and shared experiences. Since community values and practices often shape health communication, a group discussion allows us to explore a variety of views through dynamic conversations. FGDs also enable us to understand diverse views and experiences of health communication in a shorter period.

***Sample size and sampling strategy.*** Different community members have different perspectives, opinions, preferences, and levels of communication; keeping this in mind, we will conduct different FGDs. SCD is a genetic condition, and the lack of awareness about it during reproductive age significantly contributes to the increasing prevalence of traits and disease within the community. We will do FGDs with youth and adults separately to capture age-based preference and let every individual express their opinion freely. In both categories, we will conduct FGDs separately for men and women, as there is often hesitation among women and/or dominant voices of men. We will conduct multiple FGDs until a thematic saturation.

We will select participants through interactions and recommendations from local community leaders and elders at hamlets, who serve as community gatekeepers. Their familiarity with the local context and people will help select appropriate participants who can provide valuable insights.

***Participant recruitment criteria***

1. Participants must belong to the Soliga community and reside in the Chamarajanagar district.
2. Participants must be between the ages of 18 and 40 years old.
3. Participants who are willing to participate, engage and contribute in the focus group discussion.

***Procedures for data collection.*** All the expected participants will be informed two weeks in advance and invited to be part of this FGD. We will use an FGD guide to facilitate the discussions. It will also include thematic outcomes of previous scoping reviews and quantitative studies to further prompt during the FGDs. Due to the remoteness of the location of the participants, it takes around half a day for them to participate in the FGDs. There is a lack of frequent public transportation, which causes them to rely on private vehicles. Hence, we will compensate INR 300 to cover their travel expenses and a portion of their daily wage. This compensation will be paid to participants upon completion of the FGD, and they will be informed about this during the selection process.

We will record the interactions upon consent for further data extraction and analysis. An observer and a note-taker will be part of the FGD process. The researcher would moderate the session and ensure all participant's voices are heard and recorded.

***Data collection tools.*** A broad, semi-structured FGD guide has been developed based on the objective ( annexure 2). This will be further improved based on the preliminary findings from the quantitative study and the thematic outcomes of scoping.

***Consent process in detail.*** The researcher will explain the study's purpose, procedures, and potential risks and benefits clearly and understandably and obtain written informed consent. To ensure comprehension, the researcher will use locally appropriate language. Steps will be taken to avoid coercion, and participants will be assured that their participation is voluntary. In case a participant is not able to read and comprehend, data collectors will read out the consent process and about the study and, upon agreeing, will take a thumb impression of the participant in the presence of a witness.

***Data extraction and analysis.*** We will conduct a thematic analysis of our focus group data on community-preferred and culturally appropriate methods for SCD communication among the Soliga communities. We will first transcribe the FGDs. Then, we will systematically code the data, identifying key topics such as preferred communication methods, cultural elements, preferences in language and platforms and barriers to effective communication. After coding, we will organise these codes into broader themes and review the themes to ensure they are coherent and distinct. We will conduct the analysis using NVivo software. NViVo is a proprietary software for data analysis, the free alternative software for NVivo would be QualCoder 3.6 for data analysis.

## Stage 3: Evidence synthesis and guide development

In this stage, we will apply triangulation to compare and synthesise evidence and findings from the previously conducted quantitative and qualitative components of the study. From the quantitative study, we will gather insights into the existing knowledge of SCD within the selected community, the sources of information, and the available health communication-supportive infrastructure.

The evidence generated from Focus Group Discussions (FGDs) will primarily focus on community preferences for health communication, including feasible methods, preferred language, and communication platforms. In the scoping review, we will explore insights from various communication approaches attempted in Indigenous communities worldwide, highlighting their strengths and limitations.

This synthesis of evidence will inform the development of a comprehensive guide. The guide will compile all necessary information and key elements to design effective health communication strategies for the Soliga community and other similar Indian Indigenous communities regarding SCD.

### Analysis plan

The analysis plan for each stage has been detailed in the corresponding sections.

## Discussion

India's indigenous populations are diverse, with differences in culture and practices ( Victoriamma, 2018). Hence, generalising public health strategies and approaches may not be appropriate. With this diversity – health communication strategies cannot be planned and executed with a one-size-fits-all approach.

SCD is also prevalent and of public health concern among the Soliga community in Karnataka. JAGRITI study will help examine the existing SCD awareness, understand how the Soliga community perceives health communication, their health communication preferences, and how public health mechanism can re-align their communication strategies to reach this community effectively.

According to existing studies, health awareness initiatives have a significant and essential role in improving public health. Well-planned health awareness campaigns are essential to public health, supporting diagnosing, treating, and preventing health challenges. Campaigns and other forms of intervention raise community knowledge of diseases and risk factors.

For health communication campaigns to become successful among these communities, they must also resonate with the community's aspirations by being culturally appropriate and inclusive. There is a significant gap in evidence-based SCD-related health communication in the country ( Surti *et al.*, 2024). The National Sickle Cell Elimination Mission also emphasises health communication as one of the fundamental pillars, along with diagnosis and management. This study will generate new knowledge to inform the mission and help the Soliga community. The guide we aim to develop as part of the study will inform future SCD health communication campaigns and strategies for the Soliga community. It may also serve as a guide to SCD health communication in similar characteristic communities.

### Limitations of the study

The Principal Investigator (PI) belongs to a non-Indigenous community, which may influence data collection and interpretation despite efforts to engage sensitively and respectfully with the Soliga community. Cultural differences could affect how participants respond or how the PI interprets nuanced cultural contexts. Additionally, we are conducting the survey and FGDs in a single district of south Karnataka, which may limit the generalizability of the findings to larger Indigenous communities or other regions. We are developing only a guide that will help future SCD health communication, but the study does not directly benefit the community through tangible awareness materials. Since this guide is specifically SCD-focused, it may provide guidance to some extent but will not serve as a comprehensive tool for all health communication related to other diseases. Furthermore, although SCD affects non-Indigenous communities as well, the outcomes of this study may not be fully applicable to those populations due to cultural and contextual differences.

## Dissemination

The study findings will be disseminated, targeting both academic and community audiences. We will publish the study findings in peer-reviewed publications, present at conferences and on the institute website. Findings will be shared with the community along with sessions on SCD awareness (prevention, Diagnosis and Management) during the Adivasi Arogya Samvada events, an open health dialogue platform co-created by Indigenous Community-Based Organisations (CBO) of Chamarajanagar district and Institute of Public Health Bengaluru at taluk and district level in Chamarajanagar. The findings will also be disseminated at SCD-related gatherings, such as World Sickle Cell Awareness Day and other significant events where members of the Soliga community actively participate.

## Ethics and consent

The study protocol has been approved by the institutional ethics committee of the Institute of Public Health Bengaluru (Study ID 12/2024/FR vide letter number IPH/24-25/E/224 dated December 17, 2024).

As described in detail in the appropriate methods sections, we shall obtain informed written consents from respondents for the survey as well as the focus group discussions. For survey and focus group discussions, trained data collectors will conduct the informed written consent process before data collection. Participants will be approached individually in familiar community settings, ensuring privacy and confidentiality. The data collectors will use the information sheet to explain the study's purpose, procedures, and potential risks and benefits. Participants will be assured that their participation is voluntary. Consent will be documented through signed consent forms, with copies for participants' records. In case a participant is not able to read and comprehend, data collectors will read out the consent process and about the study and, upon agreeing, will take a thumb impression of the participant in the presence of a witness.

## Data Availability

No underlying data are associated with this article. Figshare: JAGRITI - Cross-sectional Survey questionnaire with Kannada translation. https://doi.org/10.6084/m9.figshare.28540640 ( Rao, 2025a) Figshare: JAGRITI Focus Group Discussion Guide with Kannada translation. https://doi.org/10.6084/m9.figshare.28540664 ( Rao, 2025b) Data are available under the terms of the Creative Commons Attribution 4.0 International license (CC-BY 4.0).
