# Supplementary material for: JAGRITI (ಜಾಗೃತಿ) - A study on enhancement of health communication for sickle cell disease (SCD) among Soliga Indigenous community in Chamarajanagar, Karnataka: Study protocol
Source: Wellcome Open Res. 2025 Jul 15;10:353. [Version 1] doi: 10.12688/wellcomeopenres.23935.1 (PMC13062772; doi:10.12688/wellcomeopenres.23935.1)
Supplement: Peer review history

# Reviewer report: Reviewer response for version 1

**DOI:** 10.21956/wellcomeopenres.26405.r152340

**Published:** 2026-04-08

**Authors:** Nirmalya Mukherjee

**Affiliations:** 1 Manbhum Ananda Ashram Nityananda Trust (MANT), Kolkata, West Bengal, India

This protocol addresses a genuine and under-researched public health problem. The burden of SCD in tribal communities is well established, and the lack of culturally grounded health communication is a real barrier to awareness and management. The study team has strong community partnerships and an ethically thoughtful approach to consent and data collection, including the decision to recruit Soliga community members as data collectors.

**What must be addressed:**

**1. **Add specific SCD prevalence or carrier rate data for the Soliga community in Chamarajanagar district (or the closest available proxy) and use this to justify site selection.

 2. Harmonise the objectives as stated in the abstract with those listed in the objectives section of the paper.

 3. Clarify the conceptual positioning of the scoping review within the mixed-methods framework.

 4. Address the exclusion of adolescents (under 18 years) more directly in the participant selection rationale.

 5. Include a short positionality statement within the FGD methods section, beyond what is currently only noted under limitations.

 6. Provide the scoping review search strategy, including at minimum an illustrative search string showing how PCC framework terms will be combined using Boolean operators across the named databases.

 7. Specify an anticipated range for the number of FGDs (minimum and maximum) and describe how thematic saturation will be determined and documented.

 8. Describe the approach to evidence synthesis in Stage 3 more concretely, including the triangulation process and any planned community validation of the guide.

 9. Consider increasing the inter-rater reliability check for the scoping review from one-in-ten to at least 20% of articles.

 10. Add a summary of FGD topic areas in the protocol text, rather than relying entirely on the Figshare annexure.

 11. Clarify whether the questionnaire used in Stage 1 has been piloted in the community and, if so, report the results of that piloting.

 Additional Comments on Language and Grammar

 The writing is generally clear and readable. The plain language summary is well done and would be accessible to community members and non-specialist stakeholders. A few specific points:

 1. The abstract uses the phrase "dearth of knowledge," which is slightly formal for a plain-language context, though it is used in the main text rather than the PLS.

 2. "Promot" in the plain language summary (page 2) is a typographical error and should read "promote."

 3. The phrase "we are incredibly grateful" in the acknowledgements is informal and can be simplified to "we are grateful."

 4. The reference "Lkreps (1988)" appears twice in the text and appears to be a misspelling of "Krebs" or possibly "Kreps." This should be checked and corrected across all instances.

 5. "Medya Pradesh" in the introduction should read "Madhya Pradesh."

 6. The statistical data on SCD awareness across states (78.4% in Gujarat, 1.1% in Assam, 10.9% in Karnataka and Madhya Pradesh) are presented without a direct in-text citation at the point of use. These figures are attributed to Babu et al. (2022) elsewhere, but the specific sentence on page 4 where these percentages appear needs an inline citation.

 7. The protocol inconsistently uses "Indigenous" (capitalised) and "indigenous" (lowercase) across sections. Given that the study team appears to prefer the capitalised form as a marker of recognition, this should be applied consistently throughout the paper.

 8. The sentence "NViVo is a proprietary software for data analysis, the free alternative software for NVivo would be QualCoder 3.6 for data analysis" reads awkwardly and contains a comma splice. This can be rewritten as: "NVivo is proprietary software used for qualitative data analysis; the free alternative considered for this study is QualCoder 3.6."

 The study has real value, and the protocol is largely sound. With the revisions described above, it would meet the standard expected for a study protocol at Wellcome Open Research

Is the study design appropriate for the research question?

Yes

Is the rationale for, and objectives of, the study clearly described?

Partly

Are sufficient details of the methods provided to allow replication by others?

Partly

Are the datasets clearly presented in a useable and accessible format?

Not applicable

Reviewer Expertise:

Public health research and practice with tribal and Indigenous communities; health communication; community-based interventions; disease prevention and awareness; evidence-based programme design in resource-limited settings

I confirm that I have read this submission and believe that I have an appropriate level of expertise to confirm that it is of an acceptable scientific standard.

# Reviewer report: Reviewer response for version 1

**DOI:** 10.21956/wellcomeopenres.26405.r137462

**Published:** 2025-11-22

**Authors:** Kapilkumar Dave

**Affiliations:** 1 Society for Education, Welfare & Action (SEWA), Bharuch, Gujarat, India

Author explained the rationale and methodology in well detail.

 There are few minor comments,

 1. Earlier studies point to generally low levels of awareness about SCD among indigenous communities. Also, awareness level varies across geographical sites in India. The highest reported in the district of Gujarat was 78.4%, and the lowest was among the districts of Assam, at 1.1%, and 10.9% in Karnataka and Madya Pradesh.- add reference. if discrepancies in method, please mention. 2. In introduction or study population please mention why population of  Chamarajanagar district was selected- what proportion and which population have high SCD prevalence.

 Thanks

Is the study design appropriate for the research question?

Yes

Is the rationale for, and objectives of, the study clearly described?

Yes

Are sufficient details of the methods provided to allow replication by others?

Yes

Are the datasets clearly presented in a useable and accessible format?

Not applicable

Reviewer Expertise:

Public health

I confirm that I have read this submission and believe that I have an appropriate level of expertise to confirm that it is of an acceptable scientific standard.
